# Advancements in Biochar for Soil Remediation of Heavy Metals and/or Organic Pollutants

**DOI:** 10.3390/ma18071524

**Published:** 2025-03-28

**Authors:** Fanyue Meng, Yanming Wang, Yuexing Wei

**Affiliations:** 1 Design Institute 5, Shanghai Municipal Engineering Design Institute (Group) Co., Ltd., Shanghai 200092, China; mengfanyue@smedi.com; 2 College of Environment and Ecology, Taiyuan University of Technology, No. 79 Yingze West Street, Taiyuan 030024, China

**Keywords:** biochar, heavy metals, organic substances, remediation, contaminated soil

## Abstract

The rapid industrialization and economic growth have exacerbated the contamination of soils with both heavy metals and organic pollutants. These persistent contaminants pose substantial threats to ecosystem integrity and human health due to their long-term environmental persistence and potential for bioaccumulation. Biochar, with its high specific surface area, well-developed pore structure, and abundant surface functional groups, has emerged as a promising material for remediating soils contaminated by heavy metals and organic pollutants. While some research has explored the role of biochar in soil remediation, several aspects remain under investigation. Fully harnessing the potential of biochar for soil contamination remediation is of critical importance. This review provides an overview of the preparation methods and physicochemical properties of biochar, discusses its application in soils contaminated by organic compounds and/or heavy metals, and examines the mechanisms underlying its interaction with pollutants. Additionally, it summarizes the toxicity assessments of biochar during soil remediation and outlines future research directions, offering scientific insights and references for the practical deployment of biochar in soil pollution remediation.

## 1. Introduction

With rapid industrialization and economic growth, significant amounts of pollutants, including organic compounds and heavy metals, have entered the soil environment through pathways such as atmospheric deposition, surface water runoff, and groundwater infiltration. Heavy metal contamination (e.g., cadmium, nickel, copper, zinc, mercury, and lead) and organic pollutants (e.g., DDT, polycyclic aromatic hydrocarbons, and hexachlorocyclohexane) are known for their mutagenic, teratogenic, and carcinogenic effects, as well as their significant bioaccumulation potential, which allows them to persist in the soil environment for extended periods and pose direct risks to human health through the food chain. As a result, the remediation of soils contaminated by heavy metals and organic pollutants has attracted significant attention from researchers.

Biochar, rich in inert carbon, is primarily produced through the pyrolysis of biomass such as crop residues, urban landscaping waste, manure, and other organic materials under low-oxygen conditions. It has significant potential for carbon emission reduction, with an estimated reduction of up to 6.6 Pg CO_2_-equivalent per year by 2050 [1]. In addition to its moderate cost and high durability, biochar offers potential synergistic benefits for food security and soil fertility, and it can persist in soils for thousands of years. The effectiveness of biochar in removing soil contaminants varies significantly depending on feedstock composition, pyrolysis conditions [2], soil characteristics, and pollutant types [3]. This variation is driven by the interactions between the biochar and soil, such as ion exchange, functional group complexation, physical adsorption, and surface precipitation. Several reviews have systematically examined the role of biochar in remediating soils contaminated with various pollutants. Zhang et al. [4] highlighted that biochar can effectively reduce the mobility and bioavailability of heavy metals and organic contaminants through mechanisms such as adsorption, ion exchange, and surface precipitation. Hou et al. [5] demonstrated that biochar can regulate soil microbial communities by providing habitats and nutrients for microorganisms, thereby enhancing microbial activity and promoting pollutant degradation. Additionally, biochars derived from different feedstocks exhibit varying performances in soil remediation. Biochar derived from crop straw and wood biomass, which are rich in lignin, cellulose, and hemicellulose, possesses a large specific surface area and abundant porosity. These characteristics make it highly effective in remediating organic pollutants. Specifically, it demonstrates strong adsorption capabilities for cationic organic pollutants and persistent organic pollutants (POPs), such as polycyclic aromatic hydrocarbons (PAHs), significantly reducing their mobility and bioavailability in soil. In contrast, biochar sourced from high-ash precursors, such as manure and sludge, has a high cation exchange capacity (CEC) and substantial mineral content. These attributes enhance its efficacy in remediating cationic organic pollutants and heavy metal contamination, offering unique advantages in contaminant immobilization and bioavailability reduction [6].

Based on the aforementioned considerations, this review provides a comprehensive overview of biochar preparation methods, its physicochemical properties, and the current state of research on its application in soil remediation. It also explores the interaction mechanisms between biochar and contaminants, including various heavy metals and organic compounds, along with potential risk analyses associated with its use. Furthermore, the review identifies future research directions for biochar in soil remediation, offering critical insights for its practical application in mitigating soil pollution.

## 2. Preparation of Biochar and Its Physicochemical Properties

### 2.1. Biochar Preparation

Biochar is a carbon-rich, low-density, and porous material produced through the thermochemical conversion of biomass under controlled conditions [7]. It can be derived from various organic materials, including plant tissues, wood chips, animal manure, and crop residues. Pyrolysis technology, an environmentally sustainable and cost-effective method for the comprehensive utilization of biomass, has been extensively studied [8]. The production of biochar not only promotes biomass recycling and the recovery of valuable resources but also offers a practical solution to the growing issue of organic solid waste generation.

**Figure 1:**
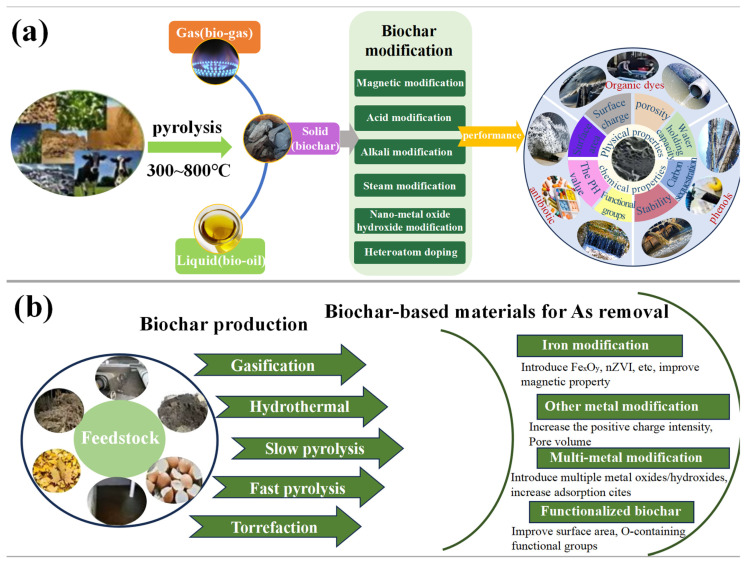
(**a**,**b**) Preparation, modification, and applications of biochar [10,11].

**Figure 2:**
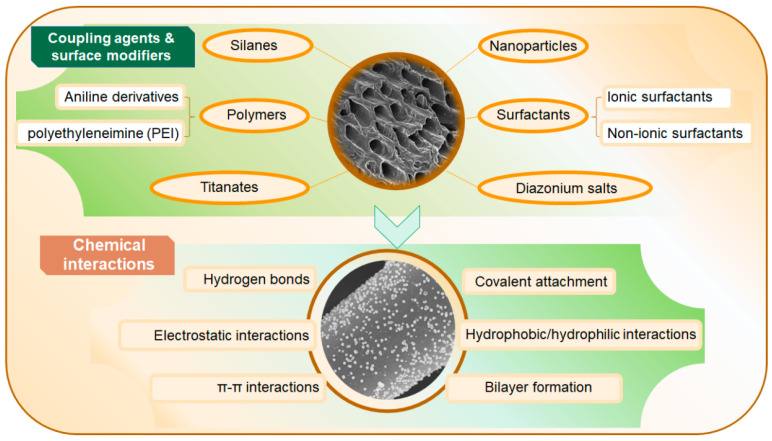
Schematic illustration of biochar surface treatment with popular coupling agents [9].

Due to the inherent limitations of biochar in adsorption and catalytic performance, extensive research has shown that its properties are largely determined by the fine-tuning of its surface chemistry. As a result, surface modification has become a crucial strategy for enhancing its performance. Marlena Geca et al. [9] primarily discussed surface modification methods for biochar, including treatment with silanes and other coupling agents such as diazonium salts, titanates, ionic/non-ionic surfactants, and nitrogen-containing molecules. These modification processes involve various reaction mechanisms, including silanization reactions, electrophilic substitution reactions of diazonium salts, and adsorption effects of surfactants. These modification techniques can alter the surface properties of biochar, such as increasing or decreasing surface functional groups, adjusting surface charge, and modifying hydrophilicity or hydrophobicity, thereby imparting new functionalities to biochar, such as improved adsorption capacity, catalytic activity, and biocompatibility. Figure 1 depicts the preparation, modification, and application of biochar. Figure 2 illustrates a schematic of biochar modification using commonly used coupling agents.

### 2.2. Physicochemical Properties of Biochar

Understanding the physicochemical properties of biochar is crucial for its effective use in soil remediation. These properties include pH, specific surface area, porosity, surface functional groups, and stability.

#### 2.2.1. pH

The pH of biochar is a crucial factor that affects its quality. Inorganic minerals and ash produced during pyrolysis, such as carbonates and phosphates, typically impart an alkaline nature to biochar. Generally, a higher pH value indicates a greater degree of biochar carbonization [12]. The surface pH of biochar is influenced by factors including feedstock biomass, pyrolysis temperature, and other conditions, with pyrolysis temperature playing a particularly significant role. As the pyrolysis temperature increases, the content of alkaline minerals in biochar rises, facilitating the precipitation and immobilization of heavy metals in contaminated soils. Conversely, when pyrolysis occurs at lower temperatures, a higher density of acidic functional groups (such as phenolic hydroxyl and carboxyl groups) forms within the biochar structure, resulting in a lower pH value [13].

#### 2.2.2. Specific Surface Area and Porosity

Kwon et al. [14] pointed out that specific surface area is a crucial factor influencing the adsorption performance of biochar, providing active sites for its interaction with pollutants. The study mentioned that different pyrolysis methods, such as steam activation, metal doping, and hydrothermal pyrolysis, can alter the specific surface area and porosity of biochar, thereby affecting its adsorption capacity. With the advancement of research, Kwon et al. [14] found that increasing the pyrolysis temperature within a certain range increases the specific surface area of biochar. However, excessively high temperatures, particularly in reactive gas media such as CO_2_, can lead to carbon volatilization, hindering pore development. This suggests that pyrolysis temperature has a significant impact on both the specific surface area and the porosity of biochar. Zhang et al. [15] further validated these findings through experiments. The researchers prepared biochar at different pyrolysis temperatures and conducted detailed characterizations. Table 1 provides a detailed list of the carbon, hydrogen, nitrogen, and oxygen contents, as well as the surface area and pore size, of biochars prepared at different temperatures (CS-300, CS-500, CS-800, MBM-500, and BS-500). The results indicate that with increasing pyrolysis temperature, the carbon content of the biochar gradually increases, while the hydrogen and nitrogen contents decrease. The surface area also increases from 3.3 to 19.6 m^2^/g. Meanwhile, significant differences in the adsorption effectiveness of biochar for different antibiotics were observed, which were closely related to the specific surface area and porosity of the biochar. For antibiotics with smaller molecular sizes, larger specific surface areas and pore volumes facilitated mass transfer, thereby improving the adsorption rate. However, for antibiotics with larger molecular sizes, the pore structure could create steric hindrance, affecting the adsorption efficiency. This clearly indicates that the relationship between specific surface area and porosity significantly influences the adsorption process, with pyrolysis temperature playing a key role in modulating this effect [14,15].

#### 2.2.3. Surface Functional Groups

The surface of biochar consists of various oxygen-containing and basic functional groups, including carboxyl, carbonyl, lactone, hydroxyl, and ketone groups, along with mineral crystals. These components serve as active adsorption sites [16]. As a result, biochar exhibits desirable properties, including adsorption capacity, hydrophilicity or hydrophobicity, pH buffering, and ion exchange. These characteristics are further influenced by factors such as pyrolysis temperature, residence time, and feedstock biomass [17].

The elemental composition of biochar reveals the presence of surface functional groups [18], with the specific types of these groups playing a crucial role in regulating the interaction between biochar and pollutants. A higher concentration of oxygen-containing functional groups on the biochar surface enhances its interaction with pollutant molecules. Research has shown that an increase in aromatic groups within biochar promotes π–π interactions with aromatic compounds in organic pollutants, thereby facilitating chemical adsorption [19]. Consequently, modification or introduction of functional groups is commonly employed to enhance the adsorption performance of biochar.

#### 2.2.4. Stability

Biochar is characterized by its low solubility, high heat resistance, remarkable stability, and strong resistance to degradation across physical, chemical, and biological processes. These properties enable biochar to maintain its stability within natural ecosystems for thousands of years [20]. Isotopic analyses reveal that approximately 97% of biochar has an average residence time (MRT) of 556 years, while the remaining 3% is considered unstable or semi-stable, with an MRT of 108 days [21]. Previous research has suggested a link between the lignin content in biomass and the stability of biochar. Specifically, higher lignin concentrations lead to the formation of more stable biochar [22].

### 2.3. Adsorption Performance of Biochar

In the field of soil pollution remediation, biochar, zeolite, and clay are commonly used adsorbents. However, biochar, with its unique properties, exhibits several advantages in terms of adsorption performance compared with zeolite and clay.

From the perspective of adsorption mechanisms, biochar surfaces are rich in functional groups such as carboxyl and hydroxyl groups, which endow it with diverse adsorption capacities. Biochar exhibits a strong affinity for heavy metals due to these functional groups, effectively immobilizing heavy metals in soil and reducing their leaching. In contrast, zeolite primarily relies on ion exchange and physical adsorption to remove contaminants, with a stronger ion exchange capacity for cations. Clay mainly adsorbs via surface charges and interlayer structures, with electrostatic adsorption and ion exchange being the predominant mechanisms. In comparison, biochar demonstrates a more diverse range of adsorption mechanisms, enabling it to more effectively address complex contamination scenarios. In soils contaminated with both heavy metals and organic pollutants, biochar can simultaneously adsorb and immobilize multiple contaminants through its various adsorption mechanisms, exhibiting a synergistic adsorption effect [23].

Regarding adsorption capacity, although zeolite generally exhibits higher cation exchange capacity and clay has some adsorption potential, their adsorption capacities are often limited by their intrinsic structure and properties. Biochar’s adsorption capacity is significantly influenced by the raw materials and preparation processes, and by selecting appropriate raw materials and optimizing preparation conditions, its adsorption performance can be significantly enhanced. Research indicates that under specific preparation conditions, biochar’s adsorption capacity for certain pollutants can exceed that of zeolite and clay. Biochar possesses a highly developed porous structure, providing a large specific surface area that offers abundant adsorption sites. The specific surface area of some biochars can rival or even surpass that of zeolite and clay, endowing biochar with strong physical adsorption capacity, enabling it to adsorb a wide variety of contaminants.

In practical applications, biochar has a positive impact on soil ecosystems. It not only effectively remediates soil pollution but also improves the soil’s physicochemical properties, such as increasing soil porosity, enhancing water retention, and improving aeration. Biochar also provides a suitable habitat for soil microorganisms, promoting their growth and reproduction, thus enhancing the ecological functions of the soil. As a soil amendment, biochar creates favorable conditions for plant growth, a benefit that zeolite and clay are relatively weak in providing. While clay has some role in improving soil structure, its effects are not as comprehensive as those of biochar, and it may even limit soil aeration. Zeolite has a less pronounced direct effect on improving soil ecosystems [23].

## 3. Biochar Used in Soils Polluted with Organic Contaminants

The primary categories of organic contaminants found in soil include organochlorine pesticides, petroleum-based hydrocarbons, phenolic compounds, polycyclic aromatic hydrocarbons (PAHs), and polychlorinated biphenyls (PCBs) [24]. Remediation techniques such as thermal degradation, steam extraction, surfactant treatment, and organic solvent extraction have been widely used. However, their potential to disrupt the soil structure and cause secondary pollution, limited applicability under specific conditions, and high costs hinder their broader use in practice. In recent years, biochar has shown promise in improving soil quality, enhancing crop yield, and removing organic pollutants from soil, demonstrating significant potential for application in soil remediation and other environmental fields [25].

### 3.1. Biochar in Pesticide-Contaminated Soils

Organic pesticides, such as insecticides, herbicides, fungicides, and nematicides, are a group of contaminants that disrupt soil microbiota. Pesticides that persist in the soil can contaminate surface or groundwater through processes like runoff, leaching, or accidental spills, thereby compromising water quality and posing risks to both ecosystems and human health [26].

Studies have shown that biochar can function as a slow-release carrier for organic pesticides, significantly mitigating their harmful effects on plants and soil [25]. When organophosphates, carbamates, and organochlorine pesticides are present together, biochar application enhances plant photosynthetic activity and boosts the activity of enzymes such as catalase, glutathione reductase, and ascorbate peroxidase [27]. Additionally, biochar can promote the growth of specific bacterial populations and facilitate the establishment of arbuscular mycorrhizal fungi, both essential for soil remediation [28]. Biochar can also modify factors like soil moisture and microbial communities, which affect pesticide degradation rates, thereby improving the adsorption and removal of pesticides [29].

Yu et al. [27] prepared two types of biochar (BC450 and BC850) from eucalyptus sawdust, using pyrolysis temperatures of 450 °C and 850 °C, respectively. These biochars were then applied for soil remediation to evaluate their effectiveness in reducing the bioavailability of two pesticides—chlorpyrifos and phorate—in onions. The results showed that biochar application significantly reduced the migration of pesticides in the soil [30]. In particular, biochar produced at the higher pyrolysis temperature (BC850) led to a marked reduction in pesticide bioavailability to plants. This effect was attributed to the strong affinity and chelation capacity of biochar for residual pesticides. Zhang et al. [31] studied the impact of biochar dosage and aging conditions on the adsorption and desorption of di(2-ethylhexyl) phthalate (DEP) in soil, using bamboo biochar. Biochar application greatly enhanced DEP adsorption in soil, with a positive correlation between the amount of biochar and DEP adsorption. Both aging processes—alternating dry–wet cycles and continuous moist aging—resulted in a decrease in DEP adsorption. Additionally, adding 0.5% non-aged biochar to soil with low organic carbon (OC) content led to a nearly 98-fold increase in DEP adsorption compared with the control. In soils with high OC content, the adsorption capacity was 3.5 times greater than that of the wet–dry cycling aging treatment and 3 times higher than that of the continuous moist aging treatment. These findings suggest that biochar application can significantly improve the soil adsorption of DEP, with the extent of improvement being influenced by both the soil’s organic carbon content and the biochar aging process. Additionally, the degradation of pesticide-related organic pollutants in biochar–soil–plant root systems has been widely studied. In these systems, the biological degradation of pesticides typically depends on factors such as soil microbial communities, microbial abundance, and the molecular structure of the pesticide [32]. The trace nutrients in biochar can stimulate microorganisms in the soil, significantly enhancing the pesticide removal efficiency. Moreover, biochar promoted more efficient electron transfer among microbial cells, pollutants, and soil organic matter, thus enhancing pollutant transformation.

In summary, numerous studies have shown that the application of biochar to soil significantly reduces the bioavailability and mobility of pesticides within the soil. Notably, while soil itself exhibits a high adsorption capacity for the pesticide Bensulfuron, the addition of biochar does not enhance this ability. This may be because biochar incorporation does not extend the retention time of low-mobility pesticides in the soil [33]. Moreover, in soils amended with wheat straw, a decrease in the bioavailability of Atrazine was observed, which resulted in a reduction in the herbicide’s effectiveness [34]. Even though pesticide residues persist in the rhizosphere for a longer period, the absorption of pesticides by plants decreases as the application dosage of biochar increases [35]. Compared with untreated soils, soils amended with 1% biochar showed significant reductions in the absorption of Furadan and Chlorpyrifos by 25% and 10%, respectively [27].

### 3.2. Biochar in Petroleum Hydrocarbon-Contaminated Soil

Petroleum hydrocarbons, including gasoline, kerosene, diesel, lubricants, paraffin, and asphalt, are common organic pollutants in the environment. These substances consist of mixtures of various hydrocarbons, such as n-alkanes, branched alkanes, cycloalkanes, and aromatic compounds, along with trace amounts of other organic compounds, including sulfur compounds, nitrogen-containing compounds, and cycloalkanoic acids [36]. The concentrations of petroleum hydrocarbons are notably higher in areas such as oil extraction and processing sites, fueling stations, petroleum spill sites, and agricultural fields near roads.

The use of biochar can significantly enhance the degradation of petroleum hydrocarbons by soil microorganisms, thereby promoting soil remediation. Wang et al. [37] used biochar derived from the co-pyrolysis of rice husks and cellulose to remediate petroleum-contaminated soils. The results showed that within a 60-day remediation period, the removal rate in the biochar-treated group exceeded 67%, which was approximately 1.4 times higher than that of the microbial control group, which had a removal rate of 46.69%. Moreover, the abundance of petroleum-degrading bacteria, such as *Proteus* spp., significantly increased, suggesting that biochar addition substantially enhanced microbial growth and degradation activity. The synergistic interaction between biochar and soil microorganisms contributed to the effective removal of petroleum hydrocarbons. Qin et al. [38] examined the effects of straw-based biochar on bioremediation and the microbial community composition over a 180-day period. Their results indicated that the degradation efficiency of total petroleum hydrocarbons (TPHs) in biochar-amended soils was notably higher than in unamended soils. The timing of biochar addition also influenced degradation efficiency, with the peak TPH removal rate (84.8%) occurring when biochar was added on day 80. At this point, a substantial amount of petroleum hydrocarbon metabolites were absorbed by the biochar, leading to a significant reduction in soil toxicity and enhanced biodegradation activity. Bushnaf et al. [39] investigated the degradation of petroleum hydrocarbons by biochar through the addition of 2% biochar to aerated sandy loam contaminated with volatile petroleum hydrocarbons. Their findings revealed that biochar addition enhanced the adsorption of petroleum hydrocarbon pollutants, while the rapid degradation of branched, cyclic, and n-alkanes accelerated the overall degradation rate. Consequently, the total amount of biodegraded pollutants was significantly higher than in untreated soils.

Moreover, due to its extensive specific surface area, biochar can act as a carrier for specialized microorganisms, promoting their incorporation into the soil to perform specific ecological functions. Guo et al. [40] successfully developed an immobilization method for petroleum-degrading bacteria on biochar, which facilitated the formation and persistence of a dominant petroleum-degrading microbial consortium. The petroleum-degrading bacteria immobilized on biochar (prepared at 500 °C, denoted as BC500-B) achieved the highest removal efficiency of total petroleum hydrocarbons (TPHs), reaching 58.31%. This removal rate was significantly higher than those observed for biochar pyrolyzed at 500 °C alone (BC500, 36.91%) and the petroleum-degrading bacterial strain 4B (43.98%). The use of biochar enhanced the content of soil organic matter, available phosphorus, and available potassium, while concurrently decreasing soil pH and ammonium nitrogen levels. Notably, the emergence of a dominant degradation community, typified by *Acinetobacter*, played a crucial role in the removal of TPHs. This research presents promising prospects for the application of interactions between biochar and bacteria in the remediation of petroleum-contaminated soils. Saeed et al. [41] immobilized two petroleum-degrading bacterial strains (Pseudomonas putida YL17 and Bacillus subtilis Pdp11) on biochar and evaluated the degradation efficiency of the microbial-loaded biochar for petroleum hydrocarbons. The results indicated that compared with the use of biochar alone, the immobilization of both bacterial strains on biochar was more effective, exhibiting a shorter half-life, enhanced biodegradation potential, and a significant reduction in the content of total petroleum hydrocarbons (TPHs) and straight-chain alkanes (C12–C18). Additionally, microbial activity was boosted. Biochar immobilized with both bacterial strains (A + B) achieved the highest hydrocarbon removal rate in the soil, reaching 67%. This was followed by biochar immobilized with strain B (34%) and strain A (29%). Compared with the control group, as well as individual biochar and bacterial strains, biochar immobilized with both bacterial strains demonstrated increases of 39%, 36%, and 41% in the activities of fluorescein diacetate (FDA) hydrolysis, polyphenol oxidase, and dehydrogenase, respectively. After immobilization on biochar, the respiration rate increased by 35% for each strain. After 40 days of immobilization, the maximum colony-forming unit (CFU/g) value reached 9.25. The synergistic effects of biochar and microbial inoculants on soil enzyme activity and microbial respiration enhanced hydrocarbon removal efficiency. Zhang et al. [42] conducted a 90-day pot experiment to investigate the potential of biochar loaded with the *Serratia* bacterial strain for remediating petroleum-contaminated soil. The findings indicated that the Serratia-loaded biochar efficiently degraded total petroleum hydrocarbons (TPHs) in the soil, achieving a degradation rate of 82.5%. During the remediation process, the activities of soil dehydrogenase (SDHA) and catalase (CAT) increased by 14% and 15-fold, respectively. In addition to the studies mentioned above, Yousaf et al. [43] investigated the combined effect of biochar and plants in the remediation of petroleum hydrocarbons, using leguminous and gramineous plants along with wood-based biochar. The results revealed that compared with biochar alone (with a total petroleum hydrocarbon degradation rate of 27%), the biochar–plant combined remediation approach resulted in a significantly higher reduction in petroleum hydrocarbons (68%). The combined application of biochar with leguminous plants led to a higher removal rate of petroleum hydrocarbons than the use of gramineous plants such as maize, wheat, and ryegrass.

In summary, biochar, as a sustainable adsorbent material and immobilization carrier, can effectively adsorb petroleum hydrocarbons in soil, preventing the diffusion of pollutants into aquatic and atmospheric environments. By adsorbing petroleum hydrocarbons, biochar also increases the surface area available for microbial interaction with the contaminants, promoting their biodegradation. While biochar alone may exhibit limited effectiveness in remediating petroleum hydrocarbons, the combined biochar–plant remediation approach shows considerable promise. Biochar enhances the growth of beneficial microorganisms in plants, making this combined approach not only environmentally sustainable but also economically viable.

### 3.3. Exploring the Use of Biochar in the Remediation of Soils Polluted by Polycyclic Aromatic Hydrocarbons (PAHs)

Polycyclic aromatic hydrocarbons (PAHs) are a group of persistent organic pollutants characterized by their fused benzene ring structures. These compounds are commonly found in soils. In China, the average concentration of total PAHs (∑16PAHs) is 2801.98 μg/kg. Biochar has emerged as a promising material for the remediation of soils contaminated with PAHs. This is attributed to its high aromatic content, well-established porosity, and large surface area [44]. Biochar also enhances the activity of bacteria that can degrade PAHs, resulting in significant changes to the composition of the bacterial community, thereby improving the bioremediation of PAHs in soils [45].

Li et al. [46] conducted a meta-analysis of 2236 observations from 56 studies to evaluate the effects of biochar preparation parameters, its physicochemical properties, soil conditions, and application strategies on the efficiency of PAH remediation. The results showed that biochar application led to an average reduction of 24.99% in PAH concentrations in contaminated soils. Biochar prepared at low temperatures was found to be effective in reducing naphthalene levels, whereas high-temperature pyrolyzed wood-based biochar was more efficient in removing PAHs with five or six aromatic rings. This research provides valuable insights and strategies for optimizing the use of biochar in remediating PAH-contaminated soils. Gomez-Eyles et al. [47] added biochar to PAH-contaminated calcareous soils for 56 days, resulting in a reduction in total PAH concentrations from 440 mg/kg to 306 mg/kg. Additionally, the bioavailable fraction of PAHs decreased from 276 mg/kg to 182 mg/kg. The study also emphasized the significant potential of biochar in soil remediation. However, the long-term impacts of biochar on pollutants and soil organisms require further investigation.

Zhao et al. [48] investigated the impacts of varying application rates (1%, 2%, and 4%) of canola straw-based biochar on PAH degradation efficiency in soil. Using enzyme activity assays, quantitative real-time PCR, and high-throughput sequencing, they also analyzed changes in soil bacterial communities and the expression of the PAH-RHDα gene. The results demonstrated that biochar addition improved the rhizoremediation of PAH-contaminated soils. Among the different application rates, 2% biochar was found to be the most effective in reducing PAH concentrations. Additionally, biochar significantly enhanced urease activity, overall bacterial abundance and activity, and the number of PAH-degrading bacteria in the soils. Some researchers have combined biochar with microbial remediation techniques, in which microorganisms with strong pollutant-degrading abilities are adsorbed onto biochar surfaces to address organic contamination in soils. Chen et al. [49] utilized biochar as a carrier for microorganisms and applied immobilization techniques to remediate polycyclic aromatic hydrocarbons (PAHs) in soil. The study revealed that biochar enhanced the mobility of PAHs in the soil, with PAHs adsorbed and accumulated on the biochar in high concentrations. These PAHs were subsequently degraded by microorganisms adhered to the biochar, resulting in a significant reduction in PAH concentrations in the soil and an increased efficiency of biodegradation. Biochar has also been employed to regulate the migration of PAHs in sludge. Hua et al. [50] investigated the impact of biochar input on soil properties, plant growth, and the migration of PAHs in soil–plant systems. Their findings showed that the application of biochar-amended composted sludge significantly improved soil buffering capacity and nutrient content compared with conventional composted sludge, thereby promoting plant growth. In two types of soil (yellow-brown soil and red soil), the biomass of ryegrass treated with biochar-amended composted sludge increased by 23%, and chlorophyll content rose by 8% and 10%, respectively. Furthermore, the addition of biochar led to a marked reduction in the transfer of PAHs from the sludge–soil system to plants. In treatments with biochar-amended composted sludge, the accumulation of PAHs in ryegrass was 27–34%, which was lower than that in conventional sludge treatments. These findings suggest that biochar, as an amendment in sludge composting, not only enhances soil properties and promotes plant growth but also effectively restricts the migration of PAHs within the sludge-soil system, thereby reducing the potential environmental pollution risks [51].

### 3.4. Biochar in Polychlorinated Biphenyl-Contaminated Soil

Polychlorinated biphenyls (PCBs) are a group of persistent organic pollutants characterized by high stability and a long-lasting presence in the soil. These compounds can be absorbed by plants, infiltrate the food chain, and accumulate in organisms, posing significant carcinogenic risks. However, there is limited research on the use of biochar to remediate PCB-contaminated soils. Most studies focus on the synergistic effects of biochar combined with bioremediation and/or phytoremediation techniques to mitigate PCB toxicity. Denyes et al. [52] examined the bioavailability and plant availability of PCBs in two contaminated soils with concentrations of 136 μg/g and 3 μg/g, respectively, by adding varying amounts of biochar. Their results revealed that when 2.8% (by mass) biochar was added, PCB concentrations in the roots of *Cucurbita pepo* ssp. *pepo* decreased by 77% and 58% in soils with PCB concentrations of 136 μg/g and 3.1 μg/g, respectively. Increasing the biochar content to 11.1% led to a further reduction in PCB concentrations in the roots of *Cucurbita pepo* ssp. *pepo*, by 89% and 83%. Additionally, PCB concentrations in the stems decreased by 22% and 54%. Furthermore, the application of biochar at 2.8% and 11.1% significantly reduced PCB concentrations in the tissues of *Eisenia fetida* by 52% and 88%, respectively. The application of biochar in PCB-contaminated industrial sites also notably enhanced the biomass of terrestrial plants and improved the survival rate of earthworms, further demonstrating the effectiveness and feasibility of using biochar for remediating real-world PCB-contaminated sites [53].

Biochar not only adsorbs PCBs molecules, reducing their bioavailability, but also effectively immobilizes microorganisms on its surface, facilitating the degradation of PCBs. Studies have shown [3,54,55,56] that the growth of PCBs-dechlorinating microorganisms requires abundant carbon sources and electrons. In the dechlorination reaction, PCBs act as electron acceptors, while biochar serves as both a carbon source and an electron donor. Microbial communities with high potential for PCBs degradation attach to biochar, forming microbial colonies that promote the degradation of PCBs accumulated within the biochar structure, thus reducing PCBs concentrations in the soil.

Additionally, biochar can enhance the stimulatory effect of plants on microbial degradation activity through two distinct pathways [57]. One of the pathways is that the plant roots can grow into the porous structure of biochar, thereby providing nutrients (e.g., root exudates) to the PCB-degrading microbial community. This can regulate the structure and function of rhizosphere microorganisms, thereby enhancing their growth and activity. Another one is that the plants can produce natural phenolic compounds, such as flavonoids, which can induce PCB degradation metabolic pathways. It was facile for biochar to bind with flavonoids, which can enhance the induction effect on immobilized microorganisms. Hayat et al. [58] employed a “biochar–plant” remediation strategy to explore plant growth, PCB degradation, and the development of PCB-degrading bacteria over a five-month period following ryegrass sowing. The goal was to evaluate the overall effectiveness of the system in remediating PCB-contaminated soil. The results revealed that PCB removal rates were significantly higher in soils with vegetation compared with non-vegetated soils. The highest removal rate, reaching 85%, was observed in the presence of biochar.

Pino et al. [59] combined PCB-degrading plants with immobilized microbial consortia to assess the impact of biochar on PCB remediation over a two-month cultivation period. The findings showed that PCB removal rates, microbial survival rates, and plant growth all reached their peak with the addition of biochar, highlighting that biochar positively affects both microbial and plant activity and metabolism.

Overall, combining biochar with appropriate bioremediation and phytoremediation techniques significantly enhances the efficiency of PCB remediation in soil, providing a promising strategy for addressing persistent organic pollutants.

## 4. Application of Biochar in Heavy Metal-Contaminated Soils

Human activities, such as mining, smelting, industrial operations, and agricultural production, significantly contribute to heavy metal contamination in soils. Contaminated soils can contain heavy metals that are typically classified into two main groups: oxygen-containing anionic metals (e.g., As and Cr) and cationic metals (e.g., Pb and Cd) [60]. The primary mechanism by which biochar and modified biochar mitigate the toxicity of heavy metals is through the reduction and precipitation of oxygen-containing anionic metals. Among various soil remediation technologies, chemical fixation is considered a straightforward, cost-effective, and environmentally sustainable approach, particularly suited for large-scale applications. Thanks to its large specific surface area and abundant functional groups, biochar enhances its ability to bind different cations. This, in turn, helps to reduce the mobility, leachability, and bioavailability of harmful elements in contaminated soils. These characteristics underscore the promising potential of biochar, particularly in terms of cost-effectiveness, performance, efficiency, and accessibility [61].

### 4.1. Immobilization of Oxygen-Containing Anionic Heavy Metals by Biochar and Modified Biochar

Unlike organic pollutants, using biochar to immobilize arsenic in soil presents certain challenges. Previous studies have indicated that biochar application slightly increases the concentration, mobility, and bioavailability of arsenic in plants [62,63]. To overcome this limitation, researchers have modified biochar with iron and its compounds (e.g., FeCl_3_·6H_2_O, FeSO_4_·7H_2_O, Fe_3_O_4_, Fe_2_O_3_, goethite, and zero-valent iron) to improve its ability to immobilize arsenic more effectively. Wen et al. [64] reported that adding 3% (*w*/*w*) of FeCl_3_·6H_2_O-modified biochar to soil reduced the arsenic concentration by 41.7%. Furthermore, arsenic levels in straw and brown rice were reduced by 61.5% and 80.1%, respectively. Similarly, Islam et al. [65] found that adding 2% (*w*/*w*) of FeSO_4_·7H_2_O-modified biochar to soil decreased arsenic content in brown rice by 29–60% and reduced the average migration rate of arsenic in rhizosphere pore water by 56%. Table 2 sums up the studies on the application of iron-modified biochar in arsenic-contaminated soils. A comparison reveals that biochar exhibits varying degrees of effectiveness and potential in arsenic remediation. Biochar derived from different sources and prepared under distinct conditions shows different arsenic adsorption and fixation capabilities. For instance, iron-modified biochar, such as that from the iron-modified Thuja occidentalis at a preparation condition of 650 °C for 2 h with 3% (*w*/*w*) FeCl_3_·6H_2_O, demonstrated a 41.7% reduction in the concentration of potentially available arsenic in soil. Concurrently, arsenic content in straw and brown rice decreased by 61.5% and 80.1%, respectively. This suggests that the addition of iron significantly enhances the ability of biochar to immobilize arsenic. In contrast, unmodified biochars, such as those from pig manure and straw, exhibited less effective remediation. Biochar from pig manure and straw, prepared at 600 °C for 6 h and modified with FeCl_3_/FeCl_2_, showed a 34% increase in arsenic leaching after 7 days of application. This could be attributed to the inherent structural and chemical properties of these biochars, which limit their arsenic adsorption and fixation capacity.

Chromium (Cr) shares similar properties with arsenic (As), both being highly carcinogenic and toxic. The primary species of chromium are Cr(III) and Cr(VI), with the toxicity of Cr(VI) being several hundred times greater than that of Cr(III) [66]. While Cr(III) is less toxic and tends to form easily removable precipitates across a wide pH range, Cr(VI) is a priority contaminant due to its higher mobility and resistance to precipitation. Consequently, it is essential to treat Cr(VI)-contaminated water and soil and convert it into less toxic forms. However, biochar, being generally alkaline, does not efficiently adsorb chromium on its own. When introduced into soil, biochar can raise the pH, which may promote the conversion of Cr(III) to Cr(VI) and enhance Cr dissolution [67]. To improve biochar’s Cr(VI) adsorption capacity, methods to increase its cation exchange capacity are crucial. Rafique et al. [68] modified biochar with N,N1-methylene bisacrylamide and acrylamide, which increased the surface positive charge and significantly enhanced its Cr adsorption capabilities. This modification reduced Cr uptake by plant roots by 65%. Similarly, Murad et al. [69] modified biochar with the cationic surfactant cetyltrimethylammonium bromide. Their results showed that the surface charge of biochar shifted from negative to positive, and the modified biochar exhibited more than a twofold increase in Cr(VI) removal efficiency compared with unmodified biochar. Medha et al. [70] utilized iron-modified biochar for the removal of chromium (Cr) from soil, achieving an effective reduction in chromium by 42.63%. Chen et al. [71] prepared Fe-BC for soil remediation, and the results demonstrated that Fe-BC was capable of removing approximately 71% of Cr from contaminated groundwater. Additionally, Cr in both groundwater and soil was effectively immobilized within the surface soil, leading to a reduction of over 81% in the leachability of Cr from the surface soil. Su et al. [72] developed a cost-effective and efficient biochar-supported zero-valent iron nanoparticle (nZVI@BC) using bagasse as the precursor through a pyrolysis process. This modified biochar was then applied for the remediation of Cr(VI)-contaminated soil. Remediation experiments indicated that the addition of 0.8% nZVI@BC to Cr-contaminated soil over a 15-day period resulted in immobilization efficiencies of 100% for Cr(VI) and 91.94% for total Cr. Concurrently, mustard growth tests revealed that nZVI@BC significantly enhanced soil fertility, reduced Cr(VI) phytotoxicity in seedlings, and promoted the growth of mustard plants.

**Table 2:** Summary of research results on the remediation effect of biochar and modified biochar on arsenic-contaminated soil.

Biochar and Modified Biochar	Preparation Conditions	Modification Agent	Soil		Addition Amount (wt. %)	Research Results	References
			**pH**	**As** **(mg·kg^−1^)**			
Rice straw biochar	400 °C, 2 h	Fe^2+^/CaCO_3_	4.65	46.89	1–3%	Ca-MBC reduced the bioavailability of As.	[62]
Oil palm fiber biochar	700 °C, 4 h	Nano zero-valent iron (nZVI)	-	-	1%	The As content in rice was reduced by 61%.	[63]
Iron-modified biochar derived from Platycladus orientalis	650 °C, 2 h	FeCl_3_·6H_2_O	5.8	141.3	3%	The concentration of potentially available As in the soil decreased by 41.7%, while the As content in rice straw and brown rice decreased by 61.5% and 80.1%, respectively.	[64]
Iron-rich corn cobs and eggshells biochar (FCEB)	450 °C, 1 h	FeSO_4_·7H_2_O	5.56	90.5	2%	The application of FCEB reduced the As content in brown rice by 29% to 60% and lowered the average As mobility in rhizosphere pore water by 56%.	[65]
Wheat straw biochar	600 °C, 1 h, 5 °C·min^−1^	Goethite	5.11 ± 0.1	2.42 ± 0.32	1.5%	The As content in the rice roots and shoots decreased by 32.2% and 46.6%, respectively.	[73]
Corn stover biochar	600 °C, 2 h	Fe-Mn-Ce	6.08–7.81	33–138	0.5–2%	Biochar reduced the bioavailable forms of As and facilitated the transformation of As from specific or non-specific binding forms into amorphous hydrated oxide forms and crystalline hydrated oxide forms.	[74]
Cedar sawdust biochar	300 °C, 2 h	Fe_3_O_4_	5.4	47.3 ± 6.7	9%	Biochar removed 20% to 30% of As within 24 h.	[75]
Wood sawdust biochar	800 °C, 1 h	Fe_2_O_3_	7.91–8.19	1900–3020	2–5%	The concentration of As in the (NH_4_)_2_SO_4_ extract decreased by 93.7% to 97.7%.	[76]
Wood sawdust biochar	800 °C, 1 h	-	7.91–8.19	1900–3020	3%	The concentration of As in the (NH_4_)_2_SO_4_ extract decreased by 31% to 56.5%.	[77]
Pig manure and rice straw biochar	600 °C, 6 h, 10 °C·min^−1^	FeCl_3_/FeCl_2_	6.3	280	2%	The application of magnetic biochar as an amendment for 7 days increased the As leaching rate by 34%.	[78]

### 4.2. The Role of Biochar and Modified Biochar in the Fixation of Heavy Metal Cations

Cationic heavy metals commonly found in polluted soils include Pb and Cd, with Pb primarily existing in forms such as Pb(OH)_2_, PbCO_3_, or Pb_3_(PO_4_)_2_. The solubility of lead in insoluble lead precipitates is enhanced under acidic conditions, which exacerbates the risks of lead contamination in acidic soils [79]. Currently, most research focuses on the immobilization of lead in acidic soils. Table 3 presents findings from several studies on the effectiveness of biochar in immobilizing Pb. For instance, Mujtaba Munir et al. [80] observed a substantial decline in the exchangeable Pb fraction, from 47% to 18%, after the application of bamboo charcoal. Sun et al. [81] found that applying 0.05–5% of shrimp-shell-based biochar reduced the effective lead content in acidic soils by 1.87–16.48% and in alkaline soils by 1.00–11.09%.

Biochar, on its own, often falls short in remediating complex Pb-contaminated soils. This limitation has driven the development of various techniques to modify biochar and enhance Pb immobilization. Naeem et al. [82] modified biochar using potassium magnesium phosphate cement, while Turan [83] used dicalcium phosphate for the same purpose. These modifications increased the PO_4_^3−^ content in biochar, improving Pb immobilization and reducing associated risks to human health and the environment. With technological advancements, researchers have also found that iron and its compounds (e.g., Fe_3_O_4_, FeCl_3_∙6H_2_O, and nano-zero-valent iron) effectively modify biochar to enhance Pb removal [84,85,86]. However, studies indicate that when modified biochar is applied to soils, it may lead to an increase in lead accumulation in plants. Iron-modified biochar has been observed to boost lead content in straw by 57.4–281.1% and in brown rice by 90.1–138.7% [64]. Similarly, sulfur-modified biochar has increased Pb content in the stems and roots of ryegrass by 55.65% and 73.43%, respectively [87].

In addition to immobilizing lead (Pb) in soils, significant research has been conducted on the use of biochar to immobilize cadmium (Cd) in contaminated soils. Table 4 summarizes findings on the effectiveness of biochar and modified biochar in Cd immobilization. The results show that biochar derived from various biomass materials significantly reduces Cd accumulation in plants. However, in some cases, the addition of biochar can also increase Cd accumulation in plants. Wen et al. [64] found that Fe-modified biochar led to an increase in Cd concentrations in straw by 169.0–390.1% and in brown rice by 263.6–268.8%. Gu et al. [88] combined corn stover biochar with Cd-contaminated soil. After a specific time frame, their analysis showed that Cd accumulation in the leaves, roots, and stems increased by 206%, 36%, and 52%, respectively.

Furthermore, the application of purely alkaline amendments to acidic contaminated soils often results in soil compaction and structural degradation. In contrast, biochar, being alkaline, provides a necessary condition for effectively altering the pH of acidic soils when applied in large quantities. Wei et al. [89] applied alkaline materials including alkali slag and biochar to soil to increase its pH. As a result, the effective Cd content in the soil decreased by 52.4–68.6%, while Cd concentrations in the plant stems and roots were reduced by 40% and 60%, respectively. Wang et al. [90] modified biochar using dimethyl disulfide and demonstrated that the sulfur-modified biochar reacts with Cd in the soil to form CdS, resulting in a 92.02% reduction in extractable Cd. Numerous studies have shown that iron and its compounds, including Fe^3+^/Fe^2+^, goethite, and zero-valent iron, are the most commonly used materials for biochar modification. The addition of these modifiers significantly enhances the efficiency of biochar in immobilizing Cd [65,91,92].

Biochar and its modified forms have shown promising results in remediating soils contaminated with cadmium (Cd) and lead (Pb). Yang et al. [93] synthesized biochar from broad bean straw and then modified it to create iron-manganese-modified biochar (FMBC), which was used to treat Cd- and Pb-contaminated soils. The results revealed that both biochar (BC) and FMBC increased the soil pH, as well as the availability of phosphorus and potassium, compared with the control group. When 5% BC was added, extractable Cd and Pb concentrations decreased by 60.98% and 51.63%, respectively. In contrast, FMBC reduced the bioavailability of Cd and Pb by 57.51–73.73% and 52.25–69.87%, respectively. Additionally, FMBC decreased the concentrations of Cd and Pb in the unstable fraction and increased their content in the residual fraction, demonstrating its ability to limit the accumulation of these heavy metals in plant tissues. In conclusion, iron-manganese-modified biochar has proven to be an effective and eco-friendly soil remediation agent with strong adsorption capacity and long-term effectiveness, making it a promising solution for mitigating heavy metal contamination in soils.

**Table 3:** Summary of the research findings regarding the remediation impacts of biochar and modified biochar on soil contaminated by lead.

Biochar/Modified Biochar	Preparation Conditions	Modification Agent	Soil		Addition Amount (wt. %)	Research Results	References
			**pH**	**Pb (mg·kg** **^−1^)**			
Cedar sawdust biochar	300 °C, 2 h	Fe_3_O_4_	5.4	12.1 ± 4.4	9%	The bioavailable Pb content was reduced by 32%.	[75]
Kitchen waste, corn stover, and peanut shell biochar	400 °C, 2 h	-	6.77	1343.8	0.6%	The extractable Pb concentrations in the soil decreased by 56.42–71.01% (KWB), 48.24–64.35% (CSB), and 43.74–60.25% (PHB).	[94]
Bamboo-based biochar	500 °C, 30 min, 10 °C·min^−1^	-	6.23 ± 1.56	25.87 ± 2.28	2%	The extractable Pb concentration in the soil decreased from 47% to 18%.	[80]
Crayfish shell biochar	300–700 °C, 2 h, 15 °C·min^−1^	-	4.50–7.85	-	5%	The bioavailable Pb content in acidic soils decreased by 1.87–16.48%, while that in alkaline soils decreased by 1.00–11.09%.	[81]
Biochar from discarded branches of bougainvillea	450 °C	Magnesium potassium phosphate cement	7.1	600	3%	The Pb concentrations in the shoots (85%) and roots (78%), as well as the extractable Pb concentration (73%), were significantly reduced.	[82]
Pistachio shell biochar	350 °C	Dicalcium phosphate	5.73	600	2%	The maximum reduction rate of DTPA-extractable Pb, as well as the Pb concentrations in the shoots and roots, reached 58%,66%, and 53%, respectively.	[83]
Coconut shell biochar	350 °C, 4 h	-	6.72	47.35	1%	The bioavailable Pb content in the soil decreased by 45.3%.	[84]
		Fe^3+^				The bioavailable Pb content in the soil decreased by 21.8%.	
Green tea biochar	400 °C, 2 h, 3 °C·min^−1^	Nanoscale zero-valent iron	6.8 ± 0.5	386	1%	The Pb immobilization efficiency increased by 57.14%.	[85]
Palm fiber biochar	550 °C	Alkali residue	4.73	621.7	10 t ha^−1^	The bioavailable Pb content decreased by 28.3–40.8%.	[86]
Green waste biochar (GWB)	650 °C, 2 h	FeCl_3_·6H_2_O	5.8	736.2	3%	The concentration of DTPA-extractable Pb decreased by 20.6%.	[95]

**Table 4:** Summary of the research findings regarding the remediation impacts of biochar and modified biochar on soil contaminated by Cd.

Biochar/Modified Biochar	Preparation Conditions	Modification Agent	Soil		Addition Amount (wt. %)	Research Results	References
			pH	Cd (mg·kg^−1^)			
Iron-modified biochar derived from sycamore branches	650 °C, 2 h	FeCl_3_·6H_2_O	5.8	0.5	3%	The application of FeBC increased the Cd concentration in rice straw by 169.0% to 390.1% and the Cd concentration in brown rice by 263.6% to 268.8% (*p* < 0.05).	[64]
Wheat straw biochar	600 °C, 1 h, 5 °C·min^−1^	Goethite	5.11 ± 0.1	2.42 ± 0.32	1.5%	The Cd content in the rice roots and shoots decreased by 42.9% and 56.7%, respectively.	[73]
Corn stalk biochar	500 °C, 4 h, 4 °C·min^−1^	-	7.90 ± 0.12	3.73 ± 0.16	5%	Cd accumulation in beetroot increased by 206%, while Cd concentrations in the leaves and roots rose by 36% and 52%, respectively.	[88]
Palm fiber biochar	550 °C	Soda water	4.73	7.49	10 t ha^−1^	The bioavailable Cd was reduced by 52.4% to 68.6%.	[89]
Rice husk-based biochar	450 °C, 2 h	Sodium dimethyldithiocarbamate (SDD)	8.56	8.78	3%	The Cd extracted by DTPA decreased by 92.02%.	[90]
Rice husk-based biochar	600 °C	-	7.67	41.02 ± 5.47	0.8%	The Cd concentration in brown rice was reduced by 74%.	[92]
		Zero-valent iron			0.8%, BC 5%, ZVI	The Cd concentration in brown rice decreased by 83%.	
		Fe^3+^				The bioavailable Cd in the soil was reduced by 38.1%.	
Wheat straw biochar (WB)	600 °C, 1 h, 10 °C·min^−1^	-	8.55	1.26	15 t ha^−1^	The Cd concentration extractable by CaCl_2_ decreased by 32.8% to 60.5%.	[96]
Rice husk-based biochar	400 °C	Acid treatment (HCl, HNO_3_, H_3_PO_4_)	7.52	30	2%	The bioavailable Cd content in the soil decreased by 87%, while Cd accumulation in the stems and rice decreased by 83.4% and 95.7%, respectively.	[97]

### 4.3. The Impact of Biochar/Modified Biochar on the Immobilization of Other Heavy Metals (Nickel, Zinc, and Copper)

Similar to Pb and Cd, Ni, Zn, and Cu are primarily present in soils as divalent cations (Ni(II), Zn(II), and Cu(II)). As shown in Table 5, unmodified biochar significantly contributes to the immobilization of these heavy metals. In a study by Zhu et al. [98], biochar modified with humic acid and wood vinegar (BHW) was synthesized. The results indicated that the incorporation of 0.1 g of BHW per kg of soil led to an immobilization rate of Ni(II) as high as 60.1%. This was attributed to the abundant organic compounds and diverse functional groups provided by humic acid and wood vinegar, which enhanced the biochar’s ability to form complexes with and adsorb Ni(II). Mailakeba et al. [99] reported that applying 15 t ha^−1^ of biochar derived from Kunai grass reduced the levels of exchangeable and acid-soluble nickel by 33–58%. Ali et al. [100] observed that the application of 2% biochar (with a Ni content of 100 mg/kg) to soil decreased the mobility of Ni by 88.9% and its extractability by 76.7%.

The use of sludge and cotton straw amendments resulted in an 18.2% reduction in bioavailable zinc in the soil and a 30.0% decrease in its accumulation in ryegrass. Researchers suggest that biochar’s ability to immobilize zinc may primarily involve cation exchange and complexation by organic ligands. A study by Ali et al. [101] found that biochar decreased zinc concentration in both the roots and young shoots of plants. Specifically, zinc levels were reduced by 36–41% in the roots and 25–31% in the young shoots. This effect was attributed to biochar’s ability to increase soil pH, its ash-related properties, and its metal adsorption capacity. Furthermore, studies by Azeem et al. [102,103] showed that biochar derived from bovine and ovine bones significantly lowered zinc concentrations in maize roots, stems, and shoots. This reduction was linked to the high phosphorus and calcium content in the biochar, which promoted the co-precipitation of zinc with phosphates and carbonates.

Tu et al. [104] investigated the impact of biochar on the immobilization of copper (Cu) in soil. They combined functional microorganisms with biochar to create biochar loaded with heavy-metal-tolerant bacteria. As a result, the content of DTPA-extractable Cu decreased from 127.3 mg/kg to 73.4 mg/kg. This reduction was attributed to the involvement of functional groups (-NH-, -COOH, -OH, and PO_4_^3−^) on the bacterial cell surfaces, which facilitated the bioadsorption and biomineralization of Cu. When 10% biochar was applied to the soil, a remarkable 96% reduction in bioavailable copper was observed. The porous structure of biochar, along with the functional groups on its surface, enhanced complexation and adsorption processes [105].

Overall, numerous studies have shown that biochar effectively adsorbs heavy metals, preventing their seepage into groundwater or absorption by plants. In addition to improving soil fertility and altering soil structure, biochar also promotes plant growth and helps reduce soil erosion. Biochar produced from different feedstocks has been found to be effective in limiting the uptake of heavy metals by plants and decreasing their bioavailability in the soil. However, the long-term effects of biochar on heavy-metal-contaminated soils still need further investigation.

**Table 5:** Summary of research results on the remediation effect of biochar and modified biochar on Ni-, Zn-, and Cu-contaminated soil.

Biochar	Operational Conditions	Modifiers	Soil				Application Rate (wt. %)	Effects	References
			pH	Heavy Metal (mg·kg^−1^)		Concomitant HMs			
Rice hull	450 °C, 2 h	Dimethyl dithio carbamate sodium	8.56		60.31	Cd	3%	Decreased DTPA-extractable Cu by 100.00%.	[93]
Imperata cylindrica	400 °C, 0.5 h, 5 °C·min^−1^	-	5.62 ± 0.24		56	-	0.75%	The biochar application significantly (*p* < 0.05) decreased exchangeable and acid-soluble Ni (58%).	[106]
Rice straw	500 °C, 2 h	-	5.3		35	-	2%	Reduced Ni mobility (DTPA) by 88.9%.	[101]
Maize straw	500 °C	-	7.9 ± 0.08		0.2 ± 0.01	-	1–5%	The application of 1–5% rates of maize straw biochar reduced the DTPA-extractable Ni by 56%.	[102]
Sewage sludge/cotton stalks (SCB)	650 °C, 1.5 h	-	7.1 ± 0.3	Zn	203.5 ± 10.2	Pb, Cu	7.5 t/ha	SCB amendment decreased the bioavailable forms of Zn in the soil by 18.2%.	[107]
Sewage sludge/cotton stalks (SCB)	650 °C, 1.5 h	-	7.1 ± 0.3	Cu	45.7 ± 3.0	Pb, Zn	7.5 t/ha	SCB amendment decreased the bioavailable forms of Cu in the soil by 34.9%.	[107]
Shell and apple tree	500 °C	-	8.4		1860	Cd	10%	Biochar reduces the concentration of Zn in roots (36–41%) and shoots (25–31%).	[103]
Sheep bone	500–800 °C, 2 h, 10 °C·min^−1^	-	8.2		981	Cd	10%	The content of Zn in maize roots (57%) and shoots (42%) was reduced.	[104]
Cow bone	500–800 °C, 2 h, 10 °C·min^−1^	-	8.46		474 ± 15	Cd	10%	Reduced the content of Zn (55% and 40%) in the maize roots and shoots.	[105]
Maize straw	400 °C, 8 h	-	7.85		247	Cd	5%	DTPA-Cu was reduced by up to 42.3%.	[108]
Rice straw	-	-	6.87		222,115	-	10%	Bioavailable Cu reduced by 96%.	[109]
Rape straw	500 °C, 2 h	Ca (H_2_PO_4_)_2_·H_2_O and KH_2_PO_4_	5.1		218.1	Pb, Cu	3%	The additions of BC, BC-Ca, and BC-K reduced Cu concentrations in TCLP extracts by 19.2%,10.7%, and 31.0%, respectively.	[110]
De-inking paper sludge	300 °C, 500 °C	-	7.80	Ni	35.5, 36.8	-	5%	Biochar significantly decreased the leached, mobile, and plant bioavailable forms of Ni.	[111]
Rice straw	500 °C, 2 h	-	5.3		35	-	1–2%	Reduced the exchangeable fraction of Ni by 59–71% when biochar is applied at 1% and 2% rates.	[110]
Digestate, cow manure, oak	500 °C, 10 min	-			298	Cd	2%	Reduced the leached Zn with 65–73% compared with the control.	[107]

## 5. The Role of Biochar in Soils Contaminated with Organic Pollutants and Heavy Metals

The remediation of soils co-contaminated by heavy metals and organic pollutants is a complex and challenging task. As previously mentioned, biochar has shown effective remediation potential in soils contaminated with both organic substances and heavy metals. However, in soils co-contaminated with multiple organic compounds and/or heavy metals, relying solely on biochar has proven to be insufficient [111,112,113]. With ongoing research, scientists have increasingly revealed the processes involved in pollutant migration, accumulation, complexation, volatilization, and degradation. Consequently, the combined application of biochar and other remediation technologies for treating soils co-contaminated by organic compounds and heavy metals has garnered significant research interest [114].

Li et al. [115] conducted a study on the joint remediation of Cd-PHE-contaminated soil using eggplant stalk biochar and ryegrass. The research demonstrated that under optimal ryegrass planting density and appropriate biochar application, both Cd and PHE concentrations in the soil were significantly reduced, while the richness of the soil microbial communities was notably increased. Shang et al. [116] carried out a 150-day pot experiment with alfalfa to assess the remediation efficiency of rice husk and its derived biochar in soils contaminated with both PAHs and heavy metals (HMs, including zinc and chromium). Shang et al. [116] also explored the effects of rice husk and its biochar on the elimination and bioavailability of PAHs and HMs within the rhizosphere, along with the abundance of PAH-hydroxylating dioxygenase genes and the bacterial community structure. The results revealed that rice husk-derived biochar was more effective than other materials in removing PAHs and immobilizing heavy metals in the co-contaminated rhizosphere soil. Over time, the abundance of PAH degradation products increased, correlating positively with PAH removal efficiency. Additionally, rice husk-based biochar stimulated microbial growth in the rhizosphere, which played a vital role in the removal of PAHs and heavy metals. These findings suggest that the combined use of biochar and plants is a promising strategy for remediating soils co-contaminated by organic pollutants and heavy metals [117].

Electrokinetic (EK) remediation is another widely used technique for the restoration of soils contaminated with both organic pollutants and heavy metals [118]. This method involves applying low-voltage direct current to contaminated soils, generating an electromotive force that facilitates the migration of soluble pollutants. EK remediation is known for its high efficiency, controllability, and broad applicability in pollutant removal [119]. However, EK remediation has some limitations, including high energy consumption. The combination of biotechnology and electrokinetic remediation (Bio-EK) helps overcome these limitations, significantly improving remediation efficiency and reducing costs [120]. In Bio-EK systems, electrokinetics plays a crucial role by enabling the migration of microorganisms, nutrients, and pollutants, while simultaneously promoting biostimulation and bioaugmentation processes [121]. The integration of a bio-permeable reactive barrier (BPRB), which uses microorganisms as fillers in the permeable reactive barrier (PRB), with EK combines the advantages of both EK and microbial remediation. It also takes advantage of the adsorption properties of the permeable reactive barrier to enhance pollutant removal. Biochar-immobilized bacteria are considered an optimal material for bioremediation in polluted soils (BPRB). Biochar not only has the capacity to remove heavy metals or organic pollutants but also acts as a carrier for exogenous degrading bacteria, thereby enhancing microbial degradation. Ma et al. [122] devised a method that utilizes biochar-immobilized bacteria to enhance electrokinetic (EK) remediation. They carried out a 25-day laboratory experiment to remediate total petroleum hydrocarbons (TPHs) (9252.51 mg/kg) and hexavalent chromium (147.17 mg/kg) in co-contaminated soils. The results demonstrated that the incorporation of biochar-immobilized bacteria into the electrokinetic (EK) system yielded the most effective remediation outcomes. In comparison with standalone electrokinetic (EK) remediation, the removal efficiency of TPH was enhanced by 22.57%, while the Cr (VI) removal rate increased by 53.15%. Furthermore, an examination of the microbial diversity within the remediation system revealed that standalone electrokinetic (EK) remediation decreased the diversity of the soil microbial community, whereas the inclusion of biochar-immobilized bacteria significantly impacted the microbial community structure and enhanced its stability. This research further validated the efficacy of integrating biochar-immobilized bacteria to boost the electrokinetic (EK) remediation of co-contaminated soils. Moreover, it demonstrated the positive influence of this approach on optimizing the microbial community structure [123].

## 6. Mechanisms of Biochar for Remediation of Contaminated Soils

**Figure 3:**
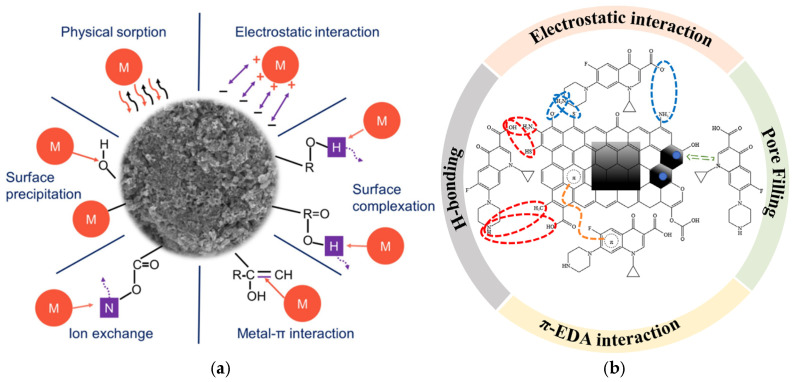
Schematic of biochar-based remediation mechanisms for contaminated soils [124]. (**a**) Heavy metal removal mechanisms. (**b**) Organic pollutant removal mechanisms.

**Figure 4:**
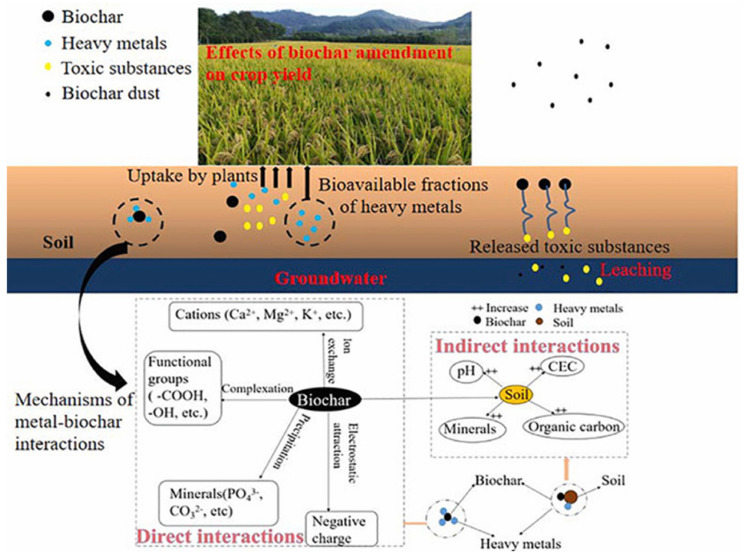
Mechanism of biochar interaction with metal ions [125].

The remediation of organic and heavy metal-contaminated soils using biochar primarily relies on adsorption and chemical interactions with surface functional groups, resulting in the transformation or immobilization/stabilization of pollutants on the biochar surface (Figure 3). One mechanism involves the formation of complexation, precipitation, and ion exchange reactions between the abundant functional groups on the biochar surface and organic pollutants and/or heavy metals, which reduces their mobility. Another mechanism is related to the unique physicochemical properties of biochar, such as adsorption enthalpy, partition coefficient, surface configuration, and porosity, which facilitate the formation of weak interactions. These interactions, including van der Waals forces, hydrogen bonds, electrostatic attractions, π–π stacking, and pore-filling processes, occur between biochar and organic pollutants or heavy metals. Consequently, the migration and diffusion of these contaminants are hindered, promoting their accumulation on the biochar surface. A third mechanism involves redox reactions on the biochar surface, triggered by various environmental factors, including light, temperature, and pH. These reactions contribute to the degradation of organic pollutants, transformation of heavy metals’ oxidation states, and reduction in their toxicity. Soil contamination is typically complex, and during soil remediation, biochar interacts with pollutants through multiple mechanisms concurrently (Figure 4).

### 6.1. Van Der Waals Force and Hydrogen Bonding

Van der Waals forces play a crucial role in the adsorption mechanism. These relatively weak interactions arise from dipole–dipole forces between molecules. According to Roy et al. [126], electronegative atoms within neutral molecules—such as fluorine, oxygen, chlorine, and nitrogen—can attract electron clouds through the formation of covalent bonds with atoms of lower electronegativity. This process enhances the dipolar character of the molecules, generating partial positive (δ+) and negative (δ−) charges. These charges form when the positive end of one molecule aligns with the negative end of another. The strength of Van der Waals forces is influenced by factors such as biochar composition, the type of adsorbate, the reaction system, and the surface roughness and geometry of both the biochar and the adsorbate [127]. These interactions are highly selective and can be characterized by the precise distance, orientation, and angle between the atoms [126].

### 6.2. Electrostatic Interactions

Electrostatic interactions exert a pivotal influence on the adsorption of ionizable substances. The efficacy of these interactions is modulated by factors including the ionic and valence radii of heavy metals, the pH of soil solutions, and the pH at the zero-point charge (pHpzc) of biochar. Incorporating biochar into soil augments its cation exchange capacity and pH, thereby intensifying the ionic interactions between heavy metal ions and soil particles. The pH of the surrounding medium exerts a substantial impact on the surface charge, degree of ionization, and conformation of the adsorbate [20]. When the pH of the medium surpasses the biochar’s pHpzc, the biochar surface acquires a negative charge, facilitating the electrostatic adsorption of positively charged adsorbates. In contrast, when the pH is lower than the pHpzc, negatively charged oxidized ions (e.g., NO_3_^−^, AsO_3_^−^, and Cr_2_O_4_^−^) are adsorbed onto the biochar surface. In the case where the biochar surface is charged while the adsorbate is neutral, electrostatic interactions become of negligible significance [128].

### 6.3. Surface Complexation

The immobilization of heavy metals in soils that have been amended with biochar predominantly takes place via the adsorption of metal ions onto the surface of the biochar. On this surface, the metal ions establish complexes with functional groups including carboxyl, hydroxyl, and amino groups [129]. Apart from binding to these functional groups, surface complexation may entail the liberation of hydrogen and other ions, and the entire process is contingent upon the pH value. These complexes are generated through chemical coordination reactions, in which the coordination compounds serve as Lewis acid–base complexes. Within such complexes, transition metal ions act as Lewis acids (electron pair acceptors) and coordinate with ligands or Lewis bases (electron pair donors) to create coordinate covalent bonds [130]. A particular form of complexation, namely, chelation, involves an organic molecule possessing multiple functional groups (multidentate) forming a complex with a central metal atom. This reaction usually occurs between metal ions and chelating agents, leading to the formation of a cyclic structure. Both coordination and chelation, as manifestations of surface complexation, represent vital mechanisms for the elimination of metal ions from biochar.

### 6.4. Ion Exchange

The surface of biochar encompasses alkali and alkaline earth metals, like Na^+^, K^+^, Ca^2+^, and Mg^2+^, which are capable of undergoing exchange with the targeted adsorbates during the adsorption process. Ion exchange reactions entail the reversible exchange of ions between the solid and liquid phases. The solid phase, functioning as the ion exchange medium, must remain insoluble in the phase where the exchange transpires. When biochar bearing cation A^+^ is introduced into a phase that contains B^+^, an ion exchange reaction between A^+^ and B^+^ is highly likely to occur. In a similar vein, anion exchange can happen when biochar has the ability to accept anions [131]. Modifying biochar with alkali metals to augment its ion exchange capacity represents an alternative strategy for enhancing adsorption performance, especially when utilizing biomass feedstocks that have a low content of alkali metals.

### 6.5. π–π Stacking and Other π Interactions

π–π stacking interactions denote the non-covalent attractions existing between unsaturated compounds, particularly those involving π-electrons. As documented by Roy et al. [126], in contrast to conventional covalent bonding, the π-orbitals of two molecules do not exhibit overlap. The researchers further indicated that two planar organic molecules, where one is aromatic and harbors π-electrons, interact through non-covalent bonds to yield complexes or stacks. Moreover, other non-covalent interactions associated with π-systems, such as n–π interactions, can make contributions to the adsorption process. Differing from π–π stacking, n–π interactions entail molecular interactions between ions that function as electron donors and electron-rich π-systems (for instance, the aromatic rings present on biochar), which act as electron acceptors.

### 6.6. Surface Coprecipitation

Precipitation happens when macroscopic entities give rise to insoluble compounds or when a chemical substance surpasses its solubility threshold. This phenomenon is usually initiated by alterations in the pH of the reaction medium or by the presence of compounds on the surface of biochar, including minerals, enzymes, and polymers. These compounds promote the precipitation of pollutants either on the surface of the biochar or within the reaction system [129]. Nevertheless, although precipitation can play a role in the removal of specific adsorbates, in adsorption experiments, it must not exceed the solubility limit, since precipitated adsorbates can no longer be efficiently removed via the adsorption process. Consequently, only the precipitation or coprecipitation that takes place on the surface of biochar actually enhances the adsorption mechanism.

### 6.7. Distribution Mechanism

The distribution mechanism in the adsorption process is similar to that in solvent extraction as both involve the separation of compounds based on their relative solubility in two immiscible phases. During adsorption, nonpolar chemicals (primarily organic pollutants) dissolve within the non-carbonized adsorbent or the organic components of biochar derived from low-temperature pyrolysis [132,133,134].

### 6.8. Pore Filling

Biochar displays a high specific surface area owing to its profuse presence of micropores and mesopores. These pore architectures augment the adsorption capacity of porous materials, as elucidated in the previously stated mechanisms. Nevertheless, larger molecules and aqueous complexes might encounter hindrance within the smaller pores, thereby resulting in less than ideal utilization of the surface area in porous materials. As per the International Union of Pure and Applied Chemistry (IUPAC) [135], micropores are characterized by having pore widths of up to 2 nm, mesopores have a range from 2 to 50 nm, and macropores possess pore widths exceeding 50 nm. Binh et al. [136] conducted an investigation into the adsorption of the herbicide 2,4-dichlorophenoxyacetic acid (2,4-D) onto biochar synthesized from corn cobs. Their research outcomes revealed that the biochar derived from corn cobs features a high density of micropores, accompanied by a relatively lower proportion of mesopores. The adsorption mechanism encompasses pore filling, hydrogen bonding, and π–π interactions.

### 6.9. Adsorption Enthalpy and Partition Coefficient

The adsorption enthalpy refers to the heat absorbed or released during the adsorption of pollutants by biochar, reflecting the thermal effects of the adsorption process. It is crucial for understanding the adsorption mechanism and determining the type of adsorption. With increasing pyrolysis temperature, the adsorption enthalpy of biochar changes. Yang et al. [7] reported that higher pyrolysis temperatures lead to a reduction in the number of acidic functional groups on the biochar surface, thereby affecting the adsorption enthalpy. The oxidation of the biochar surface functional groups increases the oxygen content, which in turn enhances the complexation process and influences the adsorption enthalpy.

The partition coefficient is used to describe the distribution of pollutants between biochar and the soil solution, reflecting the adsorption affinity of biochar for the pollutants. In biochar-based remediation of contaminated soils, organic pollutants partition in the non-carbonized portion of biochar based on differences in their solubility between water and non-aqueous phases. The high volatile matter content of biochar allows it to accommodate more organic pollutants, resulting in higher adsorption capacity [8]. For example, the high adsorption capacities of biochar derived from soybean straw, dairy products, and pig manure for pollutants such as phenanthrene and atrazine are related to the partition coefficient.

## 7. Biotoxicity Assessment of Biochar in Soil Remediation Processes

**Figure 5:**
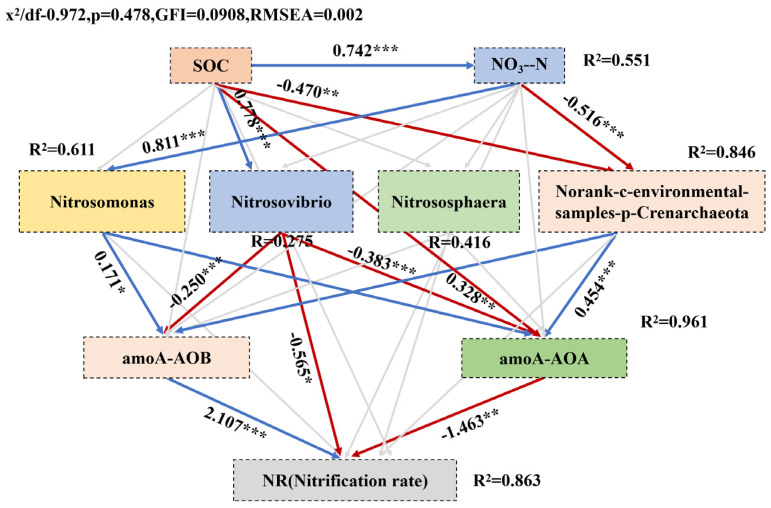
Structural equation models showing the effects of soil properties (SOC and NO_3_^−^-N) induced by biochar addition, dominant taxa of ammonia-oxidizing microorganisms, and amoA gene abundance on the soil nitrification rate (NR) at the genus level. Blue and red arrow lines indicate significant positive and negative relationships, respectively. Gray arrow lines indicate nonsignificant relationships. Numbers above arrow lines are standardized path coefficients. R^2^ indicates the proportion of variance explained by the model. *** *p* < 0.001; ** *p* < 0.01; * *p* < 0.05 [143].

Several studies suggest that biochar may inhibit the growth and metabolic activities of certain microorganisms in soil. According to Zhu et al. [137], biochar application induces oxidative stress in microbial cells through specific compounds within the biochar. This oxidative stress reduces intracellular antioxidant enzyme levels, compromises microbial cell membrane integrity, and impedes nutrient uptake, as well as the excretion of metabolic by-products, ultimately inhibiting microbial growth and reproduction. Similarly, Wang et al. [138] found that biochar application in saline agricultural ecosystems affects nitrification. The addition of biochar increases soil organic carbon (SOC) and alters the forms and concentrations of nitrogen in the soil, including NO_3_^−^-N. These changes in soil nutrient conditions may create an imbalanced or suboptimal growth environment for ammonia-oxidizing microorganisms, inhibiting their growth and reproduction. As shown in Figure 5, there is a decrease in the gene copy numbers of amoA-AOB (the ammonia monooxygenase gene in ammonia-oxidizing bacteria) and amoA-AOA (the ammonia monooxygenase gene in ammonia-oxidizing archaea). This decline indirectly indicates a reduction in the abundance and diversity of specific microbial populations following biochar application. Furthermore, biochar use may have toxic effects on plants. Xiang et al. [139] reported that soils supplemented with biochar contain higher concentrations of polycyclic aromatic hydrocarbons (PAHs) compared with non-supplemented soils. Plants can absorb and accumulate PAHs, which adversely affect their growth. For example, certain cabbage and pak choi samples exhibited PAH toxic equivalent values exceeding the maximum contamination levels, posing potential risks to human health via the food chain. Additionally, Liao et al. [140] demonstrated that transition metals present in biomass materials during pyrolysis result in the formation of polycyclic aromatic hydrocarbon-derived reactive species (PFRs). These PFRs generate highly reactive hydroxyl radicals, which significantly inhibit seed germination and plant growth.

The biological toxicity of biochar upon application is closely linked to its intrinsic composition, particularly the residual heavy metals, the action of polycyclic aromatic hydrocarbon-derived reactive species (PFRs), and the release of polycyclic aromatic hydrocarbons (PAHs) and other endogenous pollutants. Liu et al. [141] found that heavy metals released from corn cob biochar exert an inhibitory effect on soil microbial activity. Chen et al. [142] highlighted that biochar derived from rice husks and sawdust exhibits negligible toxicity toward test organisms, while biochar derived from acorus demonstrates significant toxicity to microorganisms, plants, and animals at relatively high doses. This suggests that the content and types of pollutants in biochar vary depending on its source, leading to differing toxicities. The concentration of PAHs in activated carbon depends on production conditions such as temperature and raw materials. Plant biomass contains fewer PAH precursors, and activated carbon derived primarily from plant biomass typically contains lighter PAHs like naphthalene. As pyrolysis temperature and time increase, PAH content generally decreases; however, activated carbon produced under low-temperature pyrolysis may retain higher levels of PAHs with greater bioavailability [143]. Additionally, volatile organic compounds (VOCs) and tar-like pollutants present in biochar can induce environmental stress and damage to soil upon application [144,145,146]. Activated carbon also generates PFRs during pyrolysis. Oh et al. [146] observed that PFRs present on biochar can damage cell membranes and inhibit seed germination and seedling growth. Lyu et al. [147] noted that PFRs in biochar-derived dissolved organic matter (BDOM) can trigger the generation of reactive oxygen species (ROS), which cause oxidative damage to biological cells, impair normal cell metabolism and function, and ultimately contribute to biological toxicity.

In conclusion, biochar holds potential for soil remediation, but it may also exhibit biological toxicity that could negatively affect soil microorganisms, plants, and soil fauna. This toxicity mainly arises from residual heavy metals, persistent free radicals (PFRs), the release of polycyclic aromatic hydrocarbons (PAHs), and other endogenous pollutants. Therefore, future applications of biochar in soil remediation should carefully assess these potential risks, strengthen monitoring efforts, and ensure the safety and effectiveness of the remediation process.

## 8. Conclusions and Prospects

Biochar exhibits strong adsorption ability and high reactivity toward heavy metals and organic pollutants, making it a valuable tool in environmental remediation. The primary mechanism of biochar in soil pollution remediation involves the adsorption and complexation of pollutants, which significantly reduces their mobility and bioavailability in the soil. As a result, pollutants become less accessible to crops, effectively minimizing the potential risks to human health. While biochar alone can achieve satisfactory remediation outcomes, studies have shown that combining biochar with organic fertilizers, microorganisms, or different types of biochar often yields better results than using biochar alone. Additionally, biochar modification can further enhance remediation performance when paired with other strategies, such as phytoremediation and microbial degradation. However, research on biochar’s application in soil remediation is still in its early stages. Given the variety of biomass raw materials and preparation methods used in different studies, the long-term stability of biochar in soil and its potential to pose new risks remain uncertain, and no conclusive consensus has been reached.

Given that the application of biochar in soil remediation is still in the early stages of research, a series of specific and practical research measures should be adopted to promote the development of this field. In terms of raw materials and preparation, a systematic study should be conducted on the impact of different biomass raw materials (including various agricultural and forestry waste, animal manure, etc.) and preparation conditions (pyrolysis temperature, time, heating rate, etc.) on the properties of biochar. This can be accomplished by conducting multiple comparative experiments using the controlled variable method to establish quantitative relationships between the raw material characteristics, preparation parameters, and biochar properties and to optimize the preparation process to obtain stable and efficient biochar products. To address the insufficient research on the mechanisms of action, advanced microcharacterization techniques (such as high-resolution transmission electron microscopy, nuclear magnetic resonance, etc.), combined with molecular dynamics simulations and theoretical calculation methods, should be used to deeply analyze the microscopic interaction processes between biochar, pollutants, and soil components, thereby clarifying the key mechanisms of action and their influencing factors. To make up for the lack of long-term impact studies, multiple long-term experimental sites should be established. After applying biochar to different types of soil, regular monitoring of soil physicochemical properties, microbial community structure, pollutant migration, and transformation should be conducted, with a monitoring period of no less than 10 years to comprehensively assess the long-term effects of biochar. In terms of practical application, pilot field trials and actual pollution site remediation projects should be actively carried out. For different types of pollutants (heavy metals, organic pollutants, and complex pollution) and soil conditions (texture, pH, fertility, etc.), the synergistic effect of biochar with other remediation technologies (phytoremediation, microbial remediation, chemical remediation, etc.) should be explored to optimize the combined remediation solutions. At the same time, research on organic pollutant remediation should be strengthened. Typical organic pollutants should be selected, and the adsorption and degradation characteristics of biochar should be studied. Surface modifications (such as loading metal oxides or introducing specific functional groups) and optimization of preparation processes should be used to enhance biochar’s ability to remove organic pollutants. In addition, to address the aging issue of biochar in practical engineering applications, the aging process under different environmental conditions should be simulated, and the changes in physicochemical properties before and after aging should be analyzed. Techniques to delay aging (such as surface coatings and adding antioxidants) and methods to regenerate aged biochar should be developed to reduce the risk of secondary pollution. In terms of quantitative analysis, biochar properties, soil properties, pollutant characteristics, and environmental factors should be comprehensively considered. Mathematical modeling and statistical analysis methods should be used to build quantitative prediction models for biochar adsorption capacity and remediation effects, which should be verified and optimized using extensive experimental data, thus achieving accurate evaluation and prediction of biochar’s remediation effects.

Biochar holds great potential in the field of soil remediation, yet it still faces many challenges. By implementing the aforementioned specific research directions, it is expected that the mechanisms of action of biochar will be further elucidated, its preparation and application processes will be optimized, and its remediation effectiveness will be enhanced. This will provide more effective technical support for addressing soil pollution issues and promote the widespread application and sustainable development of biochar in the field of soil remediation.

## Figures and Tables

**Table 1:** Physicochemical properties of five biochars used in this work [15].

Materials	Elemental Content (%)					BET-SA (m^2^/g)	Pore Volume (cm^3^/g)			pH_PZC_
	C	H	N	O	S		V_micro_	V_meso_	V_total_	
CS	46.24	5.98	1.97	45.42	0.07	2.6	0.0011	0.0035	0.0086	-
CS-300	55.54	4.06	1.72	37.75	0.05	3.3	0.0006	0.0040	0.0103	3.21
CS-500	57.25	2.26	1.60	37.04	0.04	4.2	0.0006	0.0041	0.0117	3.54
CS-800	59.36	1.12	1.48	36.11	0.04	19.6	0.0003	0.0059	0.0156	4.05
MBM	18.24	3.53	4.63	70.10	1.62	42.1	0.0034	0.1020	0.1250	-
MBM-500	15.08	0.96	2.07	61.34	1.13	67.3	0.0022	0.1540	0.1800	4.32
BS	66.57	6.20	0.44	36.17	0.01	4.4	0.0005	0.0002	0.0007	-
BS-500	85.74	3.12	0.41	10.33	0.01	7.2	0.0001	0.0005	0.0014	4.39

SA, surface area; V_micro_, micropore volume (pore diameter < 2 nm); V_meso_, mesopore volume (pore diameter 2–50 nm); V_total_, total volume (pore diameter 2–300 nm).
